# Exploring the Connection of Otitis Media with Effusion and Allergic Rhinitis: A Systematic Review

**DOI:** 10.1055/s-0046-1819713

**Published:** 2026-10-19

**Authors:** Álef Lamark Alves Bezerra, Gabriela Brill Ney, Fayez Bahmad Jr

**Affiliations:** 1 Instituto Santa Marta de Ensino e Pesquisa, Hospital Santa Marta, Taguatinga, Brasília, DF, Brazil; 2 Medicine Program, Centro Universitário do Planalto Central Apparecido dos Santos (UNICEPLAC), Gama, Brasília, DF, Brazil; 3 Health Sciences School, Universidade de Brasília, Brasília, DF, Brazil

**Keywords:** rhinitis, allergic, otitis media with effusion, risk factors, association

## Abstract

**Introduction:**

Otitis media with effusion (OME) is characterized by fluid accumulation in the middle ear behind an intact eardrum, often coexisting with allergic rhinitis, an inflammatory nasal condition causing symptoms such as nasal obstruction. Both conditions are prevalent in children and may share immune-mediated mechanisms linked to the *unified airway* concept. Previous studies suggest allergic rhinitis contributes to Eustachian-tube dysfunction and middle-ear effusion persistence, but the understanding of their interplay remains incomplete.

**Objective:**

To evaluate the association between allergic rhinitis and OME, identifying risk factors, diagnostic methods, and potential therapeutic strategies.

**Methods:**

The current systematic review adhered to the 2020 Preferred Reporting Items for Systematic Reviews and Meta-Analyses (PRISMA) statement. We conducted a search on the PubMed database using the Medical Subject Headings (MeSH) otitis media with effusion and rhinitis, Allergic; we identified 181 articles, which were narrowed to 15 based on the inclusion criteria.

**Results:**

Most studies revealed a significant association between allergic rhinitis and OME, particularly in children. Diagnostic methods such as intradermal dilution testing showed higher sensitivity than skin prick tests. Immunotherapy effectively addressed both conditions, even in cases with negative prick tests. Reduced micro ribonucleic acid (microRNA) 320e (miR-320e) expression was noted in allergic OME, suggesting potential as a biomarker. Surgical intervention provided symptom relief, but addressing underlying allergies proved essential. The studies were predominantly from China (20%) and the United States (13.3%).

**Conclusion:**

Allergic rhinitis plays a crucial role in OME development and symptom exacerbation. Immunotherapy may serve as a preventive strategy, emphasizing the need for clinical guidelines. The present study bridges gaps in regional medical literature, offering valuable insights for clinicians and highlighting the need for further research to optimize pediatric care outcomes.

## Introduction

Otitis media with effusion (OME) is a fluid accumulation in the middle-ear cavity, located behind a nonperforated eardrum. Allergic rhinitis is an inflammatory nasal condition that results in symptoms such as nasal obstruction. Both conditions are more prevalent in children. 1

Previous research 2 has reported that children suffering from allergic rhinitis are more susceptible to inflammatory changes in the Eustachian tube and middle-ear mucous membranes. This can lead to the development and persistence of middle-ear effusion. The idea of the “unified airway,” intimately related to the hypersensitivity mediated by immunoglobulin E (IgE), suggests that every part of the respiratory system can initiate the same inflammatory response. 3 Moreover, mast cells and their mediator, tryptase, indicate an allergic response driven by type-2 helper T cells (Th2), and are they present in most ears with chronic effusion. 4

Even though the 2017 “Treatise on Otorhinolaryngology and Cervicofacial Surgery” ( *Tratado de otorrinolaringologia e cirurgia cérvico-facial* ), by Associação Brasileira de Otorrinolaringologia e Cirurgia Cérvico-Facial 1 (ABORL-CCF) reported no significant effects on the use of antihistamines and decongestants in OME treatment, other studies 5 have observed symptomatic improvement in patients with Eustachian-tube dysfunction with nasal congestion due to allergic rhinitis when treated with oral antihistamine and nasal corticosteroids.

The current study aims to identify the association between allergic rhinitis and OME, identifying risk factors, diagnostic methods, and potential therapeutic strategies.

## Review of the Literature

**Method** — This study is a systematic review. The included studies were carefully assessed regarding study design, sampling strategies, and methodological planning in order to minimize potential bias.

Initially, the topic was chosen, and the research question was subsequently established: “Can allergic rhinitis be associated with otitis media with effusion?”. A systematic review was conducted to examine existing studies related to the chosen topic, and no literature was found. Then, the study was registered in the International Prospective Register of Systematic Reviews (PROSPERO) under number CRD42023475248.

Initially, the topic was selected and the research question was defined as follows: “Can allergic rhinitis be associated with otitis media with effusion?” A preliminary search was then conducted to identify previously published systematic reviews on this topic, and no such reviews were found. Therefore, the present study was registered in the International Prospective Register of Systematic Reviews (PROSPERO) under number CRD42023475248.

Furthermore, filtering was performed considering the criteria for inclusion (articles published from 2019 to 2023) and exclusion (articles that did not answer the research question, incomplete articles, reviews, and unfinished studies). We used the 2020 Preferred Reporting Items for Systematic Review and Meta-Analysis (PRISMA) statements to select eligible studies ( Fig. 1 ).

**Fig. 1:**
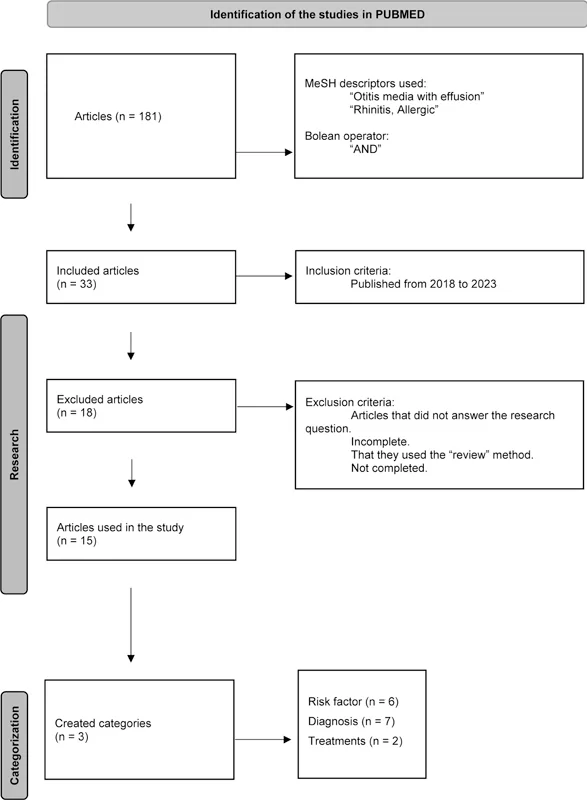
Flowchart according to the 2020 Preferred Reporting Items for Systematic Reviews and Meta-Analyses (PRISMA) statement.

The Newcastle-Ottawa Quality Assessment Scale was employed to identify biases from previous studies; each study was assessed independently by reviewers, and discrepancies in quality assessment were discussed and resolved by consensus. Additionally, peer review by two independent reviewers was performed to validate the results of the analysis.

After full-text reading, the data was collected using a specific instrument created by the main author for the analysis and discussion of the results, with the aim of composing a critical review that seeks to establish a dialogue between the referenced authors, regarding the relationship between allergic rhinitis and OME.

Initially, 181 articles were found, which were reduced to 33 after the application of the inclusion criteria, and to 15 studies 3 4 7 8 9 10 11 12 14 15 17 18 19 20 21 once the exclusion criteria were applied. This information is listed in Fig. 1 , which was developed according to a schematic adaptation by other authors. 6 Among the studies found, most were published in 2021 (33.33%; *n* = 5), followed by 2020 and 2022 (20%; *n* = 3), and 2019 and 2023 (13.33%; *n* = 2).

Regarding the countries in which the studies were conducted, most were from China (20%; *n* = 3), followed by the United State (13.33%; *n* = 2), and Iran, Nigeria, Malaysia, Indonesia, Egypt, Poland, Turkey, South Korea, India, and Italy, each with 1 study (6.66%). Table 1 shows that most articles were on cohort studies, either prospective (40%; *n* = 6) or retrospective (40.00%; *n* = 6), followed by case-control studies (20.00%; *n* = 3).

**Table 1:** Main characteristics of the included articles

Author (year; country)	Number of patients; age (years)	Study design	Purpose	Relevant results	Newcastle-Ottawa Scale score
Sharifian et al. 14 (2019; Iran)	89; 3–15	Case-control	To evaluate the correlation between AR and OME.	AR may play a significant role in the development of OME.	3
Tukur et al. 15 (2022; Nigeria)	260; 4–12	Case-control	To establish whether there is a higher prevalence of OME in the child population with AR.	Children with AR are at an increased risk of developing OME.	6
Norhafizah et al. 7 (2020; Malaysia)	130; 3–15	Prospective cohort	To define the prevalence of AR in children with persistent otitis media, as well as to identify the risk factors, common allergens. and hearing threshold in children affected with OME.	There was a 52.3% prevalence of AR. Furthermore, this study revealed that AR represents a substantial risk factor in the development of persistent OME ( *p* < 0.0001).	6
Hurst and McDaniel 3 (2021; United States)	371; 6–73	Retrospective cohort	To compare the clinical relevance and advantages between TDIs and SPTs in adults and children using immunotherapy, with suspected manifestations of allergy: chronic AR, asthma and/or chronic OME.	The IDT demonstrated greater sensitivity than the SPT ( *p* < 0,01). And it was possible to validate the strong association between allergy manifestations and OME. Through IDTs, it was possible to observe that the use of immunotherapy was able to resolve chronic middle ear diseases, such as OME.	5
Restuti et al. 12 (2022; Indonesia)	92; 5–13	Case-control	To determine whether laryngopharyngeal reflux, AR and adenoid hypertrophy represent risk elements for the occurrence of OME.	There was no statistically significant difference in the occurrence of AR between the groups with and without OME (OR: 1.62; 95%CI: 0.61–4.28; *p* < 0.05).	6
Saad et al. 8 (2020; Egypt)	310; 1–17	Cohort	To identify potential risk elements for the recurrence of serous otitis media.	A significant association was observed between AR and serous otitis media (OR: 14.27; 95%CI: 7.32–49.85; *p* < 0.0001).	5
Adamczyk et al. 17 (2021; Poland)	54; 3–9	Cohort	To analyze whether the expressions of 5 miRNAs (miR-223–3p, miR-451a, miR-16–5p, miR-320e, and miR-25–3p) show differences between children presenting OME and allergy and those who are not allergic.	The considerably-reduced expression of miR-320e in allergic patients with OME indicates a greater propensity for bacterial invasion due to impairment of the epithelial tissue ( *p* = 0.049).	5
Chen et al. 9 (2023; China)	511; < 12	Retrospective cohort	To examine the elements that affect the incidence of OME in children who have adenoid hypertrophy.	A statistically-relevant association was identified between AR and OME (OR: 1.906; 95%CI: 1.183–3.071; *p* < 0.05).	7
Zhang and Fu 20 (2022; China)	318; 1–13	Cohort	To investigate and examine the therapeutic approaches and impacts of OME in children with varying levels of hearing loss.	The efficacy rates in the drug treatment, myringotomy, and tympanotomy-tube insertion groups were of 95.6%, 89.6%, and 90.9% respectively. A multivariate logistic regression analysis revealed that AR (OR = 0.073; 95% CI: 0.013–0.401; *p* = 0.003) was the factor that influenced the therapeutic response to OME.	6
Songu et al. 10 (2020; Turkey)	539; 2–14	Retrospective cohort	To identify the main risk factors for OME in children suffering from adenoid hypertrophy.	AR was considered a risk factor for OME ( *p* < 0.001).	6
Yeo et al. 11 (2021; South Korea)	17,660; 20–70	Retrospective cohort	To examine the probability of OME occurrence in patients with gastroesophageal reflux disease, and to identify other medical conditions that may increase the risk of this condition.	A higher risk of developing OME was observed in AR patients (OR: 1.69; 95%CI: 1.37–2.10), asthma, and chronic rhinosinusitis.	7
Sidam et al. 18 (2023; India)	126; 5–18	Cohort	To analyze the connection and factors that influence the relationship between AR and ear problems, in particular, OME.	Treating AR to prevent middle-ear complications ( *p* < 0.05). A comprehensive approach that includes clinical examination, tympanometry, audiometry, nasal swab analysis, eosinophil count, IgE levels, and skin testing, along with comprehensive medical treatment, is recommended for the care of children with AR.	6
Ameli et al. 19 (2019; Italy)	1,002; 3–10	Retrospective cohort	To analyze whether certain clinical information and endoscopy findings can serve as anticipatory indicators of AR in children during an otolaryngology consultation.	Out of the 547 children with AR, 391 did not have OME, 114 had it (RR: 0.94; 95%CI: 0.69–1.29), and 39 were currently suffering from this condition (RR: 0.55; 95%CI: 0.35–0.85; *p* = 0.4498).	7
Hurst et al. 4 (2021; United States)	110; 3–70	Retrospective cohort	To evaluate the hypothesis that the IDT has superior sensitivity compared with the SPT to identify clinically-relevant allergens in patients with OME or eustachian tube dysfunction.	The use of the IDT, in cases of negative SPT, doubled the diagnosis of allergies ( *p* = 0.004).	6
Chen et al. 21 (2021; China)	70; > 17	Cohort	To evaluate whether endoscopic paranasal-sinus surgery has a positive effect on the recovery of Eustachian-tube function in patients with chronic rhinosinusitis and Eustachian-tube dysfunction, as well as to investigate the factors that may be related to this improvement in auditory function.	Patients diagnosed with OME experienced substantial improvement in the scores on the 7-item Eustachian Tube Dysfunction Questionnaire within 8 to 12 weeks after surgery ( *p* < 0.001). However, the presence of AR has been identified as a factor associated with lack of improvement after surgical intervention.	6

**Abbreviations:** AR, allergic rhinitis; IDT, intradermal dilution test; IgE, immunoglobulin E; miRNA, micro ribonucleic acid; OME, otitis media with effusion; OR, odds ratio; RR, relative risk; SPT, skin prick test.

**Risk factors –** There is a significant preponderance of allergic rhinitis in OME patients. 7 8 9 10 11 Furthermore, children with OME and allergic rhinitis present greater degrees of hearing loss than children without this association. Children with otitis media due to persistent effusion presented a higher prevalence of allergic rhinitis. 7 However, only one study 12 concluded there was no substantial distinction in allergic rhinitis between the OME and non-OME patients. Moreover, age appears to act as an effect modifier: children aged ≥ 6 years with allergic rhinitis are 4 times more likely to develop OME, whereas no significant association was observed in children younger than 6 years. 13

**Diagnosis –** In a study 14 involving 37 children with OME and 52 children as controls, the prick test confirmed the presence of allergic rhinitis in (9 subjects in the case group, and 17 OME children had at least 1 positive skin prick test to any antigen. Regarding serum levels of IgE, eosinophils and eosinophils in nasal smears, no significant differences were observed between the cases and controls; however, differences were noticed when examining neutrophils, granulocytes and bacteria. Therefore, children with allergic rhinitis should undergo routine screening due to the increased risk of developing OME. 15

Although the intradermal dilution test (IDT), a method that involves injecting a small amount of allergen under the skin and observing the skin's reaction, can yield a false-positive result, it should be performed when there is a strong suspicion of allergy and the skin prick test is negative, as the IDT has demonstrated higher diagnostic sensitivity in such cases. 16 Immunotherapy improves allergic rhinitis and OME. About 45% of OME patients had allergies to inhalants alone, and 55%, to inhalants and foods. 16 In two studies, 3 4 all patients showed improvement with immunotherapy, even those with a negative prick test and a positive IDT only at high concentrations. There was no significant difference in the response between highly- and weakly-sensitized patients, neither between those with positive prick test and negative prick test with positive IDT. Intradermal dilution tests followed by a negative skin prick test doubled the number of patients who required treatment with immunotherapy and increased the diagnosis rate by 58%.

There is significantly lower expression of micro ribonucleic acid (microRNA) 320e (miR-320e) in allergic patients with OME when compared with non-allergic patients with the same pathology. This could serve in the future as a potential aid for a biomarker or therapy. Furthermore, miR-320e stimulates the immune response, and it is capable of activating genes responsible for bacterial invasion, improving the inflammation process. 17

A study 18 reported that nasal-smear eosinophils were found more often in the participants with allergic rhinitis, with sneezing and chronic otitis media being the most common manifestations associated with them, followed by Eustachian-tube dysfunction; no correlation was found between nasal cytology and the severity of nasal symptoms or middle-ear dysfunction. The diagnosis of allergic rhinitis should be based on clinical examination, documented sensitization, such as IgE production, and proven consistency between exposure to sensitizing allergens and immediate symptom occurrence. 19

Surgical procedures to reestablish aeration can provide symptomatic relief in OME cases, but it is important to note that curing the underlying condition requires a comprehensive understanding of the pathophysiology. The middle ear, like other areas of the respiratory tract, is capable of mounting an inflammatory response, and this similarly underscores the importance of addressing the root causes of the condition. 4 The number of negative prick tests may explain why most otolaryngologists do not consider treating allergy as a possible etiology of OME. A study 4 reported that strongly- and weakly-sensitized patients showed equal improvement in OME after immunotherapy; in the OME cases, it was essential to evaluate the patient's allergic status, and in cases of confirmed allergy diagnosis, immunotherapy was an effective treatment. 4

**Treatment** – For OME, the treatment should also be tailored based on the degree of hearing loss. Miringotomy is recommended in cases of history of chronic severe hearing loss. Children with allergic rhinitis should receive appropriate medication. 20 Allergic rhinitis was identified 21 as a contributing factor in the failure to achieve normalization of the score on the 7-item Eustachian Tube Dysfunction Questionnaire (ETDQ-7) following sinus surgery in patients with rhinosinusitis and OME who underwent endoscopic sinus surgery.

## Discussion

The connection between allergic rhinitis and OME is complex and multifaceted. Both conditions share the same airway in the upper respiratory tract and are subject to similar immune responses. However, the exact nature of this relationship and how it manifests in terms of diagnosis and treatment is not yet completely understood. 1

The present study has no conflict of interest. Through a critical analysis of the available literature, we sought to provide valuable insights for healthcare professionals and researchers and to contribute to a better understanding of the relationship between allergic rhinitis and otitis media with effusion. Furthermore, by examining the literature available and conducting detailed analyses, we hope to provide valuable insights for healthcare professionals and researchers.

We will now explore the results of the present review, as well as the clinical implications and gaps that still need to be filled in understanding the relationship between allergic rhinitis and OME.

Comprehensive knowledge of modifiable risk factors could help to minimize the complications of OME. Therefore, understanding the associations between these two conditions is of utmost importance in the clinical context, as it can have significant implications for the diagnosis, treatment and well-being of affected children.

First, early identification of children who present with allergic rhinitis and OME may be essential for effective treatment. Symptoms shared between these conditions, such as nasal obstruction and hearing problems, can be mistakenly attributed to a single condition, leading to incorrect diagnoses and ineffective treatments. Therefore, understanding how these conditions are related can improve diagnostic accuracy and direct therapeutic efforts more appropriately.

Furthermore, the relationship between allergic rhinitis and OME has implications for the clinical management of these conditions. Knowing that a child with allergic rhinitis has an increased risk of developing OME, clinicians can adopt more proactive prevention and monitoring strategies. This may include considering treatments for allergic rhinitis that may also benefit the child's hearing health.

Research into this association may also open doors to new therapeutic approaches. Studies 22 have shown that intranasal corticosteroids, which are effective in treating allergic rhinitis, are not indicated and do not improve the treatment of isolated OME. Other studies 23 also argue against the use of allergy medications for the treatment of OME, warning against the use of anti-inflammatory drugs and nasal decongestants. In addition, they recommend surgical intervention with tympanostomy-tube placement for persistent cases and in children at risk of developing hearing loss.

Therefore, by exploring and understanding the relationship between allergic rhinitis and OME, the current research contributes to scientific knowledge and has a practical and direct impact on clinical practice. By recognizing the clinical relevance of these associations, we can improve the health and well-being of children and their families.

The middle ear is an immune-competent organ that is structurally connected to the Eustachian tube and the upper respiratory tract. Based on the unified airway concept, antigens that stimulate the nasal mucosa can also trigger mucosal immune responses in the Eustachian tube and middle ear. Local immune dysregulation decreases adenoid barrier function and may contribute to otitis media with effusion. 24

Immunoglobulin E, interleukin-4 (IL-4), histamine, and eosinophil levels in middle-ear effusions are increased in OME patients. In subjects with allergy rhinitis, IL-4, interferon-gamma (IFN-γ), and histamine secretions in the nasal mucosa are increased. 25

It is important to consider that most of the research is based only on test results, which often yield false-positive or false-negative results. Although in one study 14 there was a significant preponderance in the incidence of allergic rhinitis between the OME and control groups, it is important to note that this trend was not observed in all studies, as indicated by Restuti et al. 12 However, this study was the only one to confirm the diagnosis of allergic rhinitis exclusively based on a questionnaire, called International Study of Asthma and Allergies in Childhood (ISAAC), which may present a greater possibility of bias. Furthermore, Restuti et al. 12 considered only tympanogram B to be positive, but other studies 15 consider tympanogram C as well.

Another study 13 demonstrated that the association between OME and allergic rhinitis depends on the age group: children aged ≥ 6 years with allergic rhinitis are 4 times more likely to develop OME; however, no significant association was found in children under that age. Therefore, this variation in results across studies suggests the need for further analysis and consideration of additional factors.

To diagnose allergies in children with OME, it is recommended to perform an IDT with a more concentrated antigen in the presence of a negative prick test. 4 Patients with negative results on the radioallergosorbent test (RAST) or prick test are currently diagnosed as having nonallergic rhinitis with eosinophilia syndrome, 1 but a study 26 showed that 60% of the patients with this diagnosis responded to immunotherapy, based on the IDT.

For OME diagnosis, pneumatic otoscopy and tympanometry are essential exams. The audiological evaluation is reserved for cases in which the effusion persists for more than 3 months or for children at risk of presenting development delays, such as impaired speech progression. 23 However, a guideline 27 suggested that pneumatic otoscopy alone is considered the gold standard to diagnose OME, while tympanometry is reserved for uncertain cases. Unfortunately, there are no publications from Brazil or Latin America on this topic, so we recommend that future studies should be conducted in these areas.

## Final Comments

Allergic rhinitis has been demonstrated to play a significant role in the development of and exacerbation in the symptoms of OME, which can lead to complicated and/or persistent cases of this condition.

The current study significantly contributes to the knowledge of parents, pediatricians, otolaryngologists, allergists, and primary-care professionals, providing a detailed understanding of the factors associated between allergic rhinitis and OME. Furthermore, it highlights the need to implement guidelines to avoid the risk factors and, perhaps, initiate immunotherapy as a primary prevention measure for OME in children with allergic rhinitis.

We may conclude that there is a gap in the Brazilian and Latin American medical literature regarding the interaction between these two health conditions that are so common in children. Therefore, the current publication helps to bridge a void in the regional medical literature, providing valuable information for clinicians, researchers, and healthcare professionals working with children with OME and allergic rhinitis. Ultimately, the present study fills a knowledge gap and has the potential to positively impact children's health in the region, demonstrating the crucial importance of localized and relevant research.
